# Use of Consonant Evaluation for the Adjustment of a Maxillofacial Prosthesis in a Mandibulectomy Patient: A Case Report

**DOI:** 10.7759/cureus.83600

**Published:** 2025-05-06

**Authors:** Mariko Hattori, Junichiro Wada, Mai Murase, Yuka Sumita, Noriyuki Wakabayashi

**Affiliations:** 1 Department of Advanced Prosthodontics, Institute of Science Tokyo, Tokyo, JPN; 2 Department of Partial and Complete Denture, The Nippon Dental University, School of Life Dentistry at Tokyo, Tokyo, JPN

**Keywords:** adjustment, consonant evaluation, denture, mandibulectomy, maxillofacial prosthetics, speech

## Abstract

This case highlights the use of acoustic analysis in resolving speech difficulties encountered during mandibular prosthesis fabrication. Following mandibular resection and reconstruction, a 45-year-old male patient experienced persistent issues pronouncing the /sa/ consonant with a removable prosthesis in place. Psychoacoustic analysis revealed reduced sharpness in articulation, leading to precise adjustments of the prosthesis’s lingual surface. This restored speech clarity, and the patient had no further complications over six years of follow-up. This case underscores the impact of mandibular prostheses on speech and highlights the value of acoustic analysis for guiding precise prosthesis adjustments to address pronunciation difficulties.

## Introduction

Patients requiring maxillofacial prosthetic rehabilitation frequently experience significant speech impairments, making speech evaluation a fundamental aspect of treatment [1]. Previous research has focused on the objective evaluation of speech in these patients, with particular emphasis on vowel evaluation [2]. Not only vowels but also consonants play an important part in speech, and it is therefore important to evaluate consonants in the clinical situation.

Psychoacoustic evaluation, pioneered by Zwicker et al. in the 1960s, considers the human ear’s audible spectrum, masking effects, and other auditory characteristics [3]. This approach is widely used in sound design analysis across various fields, including the evaluation of sounds produced by industrial products such as cars and household equipment. Among psychoacoustic parameters such as loudness, sharpness, and roughness, sharpness has proven particularly useful for analyzing consonants [4,5]. Sharpness is the ratio of high-frequency to low-frequency components [4,5]. In the Japanese language, /s/ exhibits higher sharpness compared with other consonants [5]. In patients with maxillectomy, a reduction in sharpness during the articulation of /sa/ has been observed, which improves with maxillary prostheses [4].

Furthermore, drawing from previous research, speech evaluations have been applied in individual cases to support the design and adjustment of prostheses [6,7]. To date, most assessments of speech production have predominantly focused on maxillary prostheses [8-10]. Many consonants have their place of articulation on the palate or maxillary gingiva, so it is true that the maxilla plays an important role in speech sound production. However, unlike the maxilla, the mandible is mobile during speech, making it a key contributor to articulation as well. It supports the movements of the tongue and lower lip, both of which are essential for producing many consonant sounds. Emerging evidence suggests that the configuration of major connectors in mandibular bilateral prostheses may also influence speech [11]. Considering the tongue’s contact with the teeth and gingiva of the mandible during speech, it is plausible that mandibular prostheses could significantly affect pronunciation.

This study reports the outcomes of mandibular prosthesis adjustments following ameloblastoma resection and reconstructive surgery. The adjustments were guided by the results of an acoustic consonant analysis, leading to successful functional outcomes.

## Case presentation

**Figure 1:**
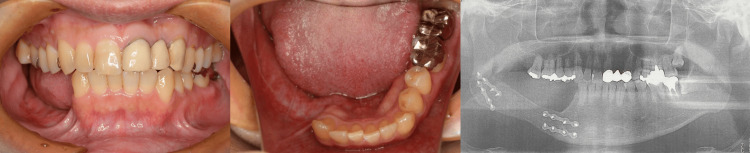
Intraoral view and the panoramic X-ray of the patient Right mandibular premolars and molars were missing. Mandibular positioning showed no deviation.

The patient was a 45-year-old man who was diagnosed with ameloblastoma in the right mandible and underwent resection of the affected region in March 2017, followed by reconstruction using a free scapular osteocutaneous flap. After six months of postoperative follow-up, he was referred for prosthodontic rehabilitation. The patient presented with missing right mandibular premolars and molars, but an occlusal examination revealed no significant abnormalities, and mandibular positioning showed no deviation. A panoramic X-ray confirmed successful mandibular reconstruction using an osteocutaneous flap (Figure 1). The patient was diagnosed with functional impairment due to missing teeth and a mandibular defect.

**Figure 2:**
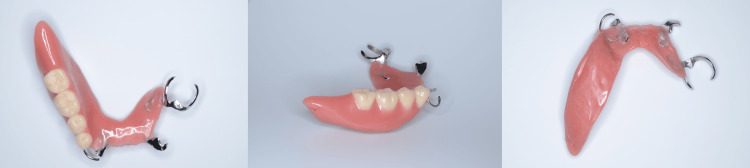
The mandibular prosthesis fabricated The prosthetic design included hard acrylic artificial teeth, a cobalt-chromium Akers clasp for the left mandibular first molar, a wired clasp with a rest for the right canine, a hook for the left first premolar, and an acrylic base.

**Figure 3:**
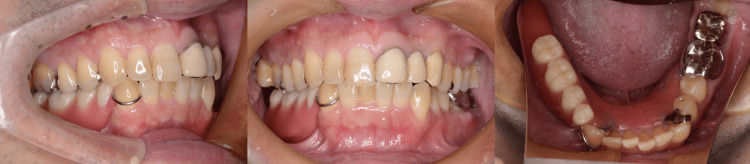
Intraoral view with the prosthesis The prosthesis was inserted, and its mucosal adaptation and occlusal relationships were confirmed to be accurate.

Prosthodontic treatment involved fabricating an acrylic-based mandibular prosthesis. After a preliminary design, a definitive impression was made using an individual tray made of tray resin (Ostron, GC, Tokyo, Japan) and silicone impression material (Examix Fine Regular, GC) with a selective pressure technique, and occlusal registration was completed using an occlusal record base. The prosthetic design included hard acrylic artificial teeth (Endura Posterior, Shofu, Kyoto, Japan), a cobalt-chromium Akers clasp for the left mandibular first molar, a wired clasp with a rest for the right canine, and a hook for the left first premolar. An acrylic base was selected in consultation with the patient, due to its ease of adjustment and coverage under the national health insurance, making it a suitable choice as the initial prosthesis after surgery. Following a wax denture trial and flange modifications using soft wax (soft plate wax, GC), the final prosthesis was fabricated using heat-polymerized denture base acrylic (Acron Dark Pink, GC) (Figure 2). At delivery, the prosthesis's mucosal adaptation and occlusal relations were confirmed to be accurate using pressure indicating paste (PIP Paste, Mizzy, NZ, USA) and articulating paper (Articulating Paper Red, GC) (Figure 3).

**Figure 4:**
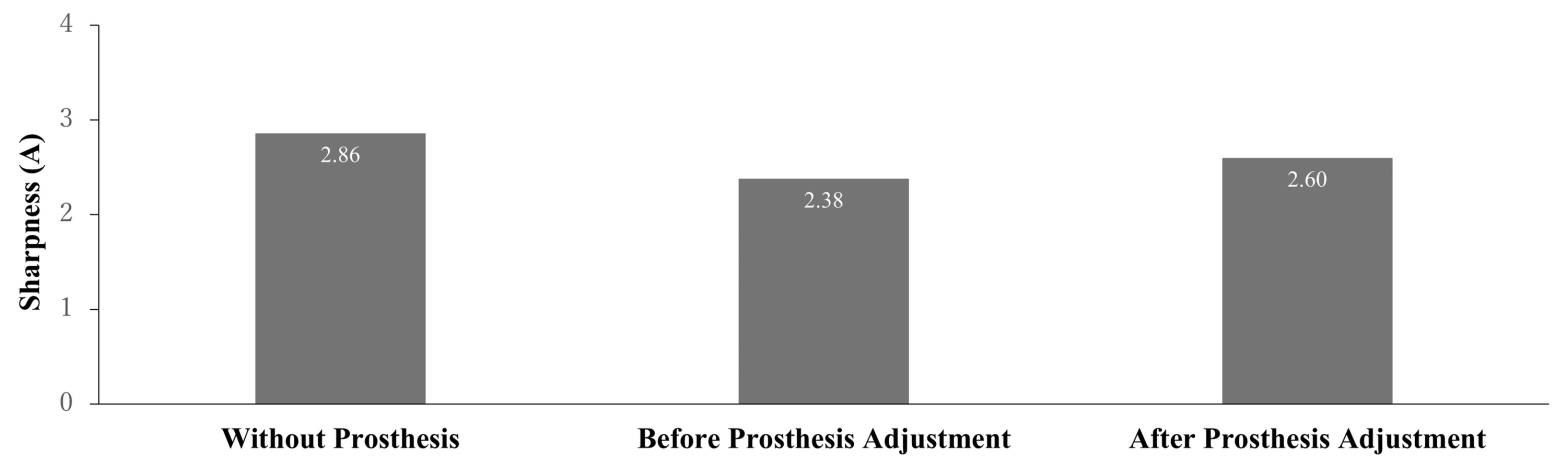
Result of the consonant evaluation Sharpness values in Acum (A) for /s/ were compared under three conditions: without wearing the prosthesis, with the prosthesis before adjustment, and after adjustment. The sharpness of /s/ was lower when wearing the prosthesis before adjustment, but returned closer to the baseline value after the adjustment.

Despite no changes in the occlusal vertical dimension or anterior tooth alignment, the patient reported difficulty pronouncing the /s/ sound, which persisted after two weeks of wearing the prosthesis. The patient sat with his lips 5 cm from a microphone, and three utterances of /asa/ were recorded on a computer in a soundproof room using sound collecting software (Osreco, Ono Sokki, Kanagawa, Japan). The highest sharpness of each utterance was measured using a psychoacoustic system (Oscope II, Ono Sokki) [5]. Acoustic analysis revealed reduced sharpness of the /s/ sound when wearing the prosthesis, suggesting restricted tongue movement. The superior aspect of the lingual denture base, extending from the lower left canine to the lower right canine, was adjusted by thinning with a carbide bur and polished using a silicone point of the lingual base using a carbide burr and polishing it with a silicone point. The consonant was evaluated again, and the improvement of sharpness was confirmed (Figure 4). Sharpness values in Acum (A) for /s/ under three conditions - without the prosthesis, with the prosthesis before adjustment, and after adjustment - were 2.86, 2.38, and 2.60, respectively. The sharpness of /s/ was lower with the prosthesis before adjustment, but returned closer to the baseline value after the adjustment. The patient was satisfied with his speech. He continued to visit the clinic twice a year to check the prosthesis and the condition of the remaining teeth. At the time of writing this report, he has worn it without issues for six years.

## Discussion

Generally, although wearing a maxillary prosthesis can significantly impact speech, the association between a mandibular prosthesis and speech functionality is considered to be minimal [12]. In maxillary prosthesis cases, it is often observed that speech functionality improves with a prosthesis in comparison to without a prosthesis [8-10]. For this reason, the majority of studies involving prostheses and speech functionality have focused on maxillectomy patients with a maxillary prosthesis, and there are few reports assessing the relationship between mandibular prostheses and speech functions. Moreover, while numerous studies on conventional prostheses have centered on speech impediments resulting from wearing a prosthesis, research concerning the relationship between maxillofacial prosthetics and speech functionality has often concentrated on improvements in speech functions from the use of maxillofacial prosthetics. Consequently, in instances like the present case, where the fitting of a mandibular prosthesis resulted in a speech impairment, previous findings that could be referenced for guidance were lacking.

Within the scope of mandibulectomy cases, including the present case, the possibility exists that alterations in the motility of the tongue, a soft tissue entity, may arise as a consequence of mandibulectomy. Therefore, consonant evaluation was used to analyze the sharpness of syllables that the patient reported as challenging to pronounce. The findings indicated a potential hindrance to tongue movement linked to the mandibular prosthesis. Following this, it was found that appropriate adjustments to the prosthesis ameliorated the patient’s articulatory difficulties.

It is commonly known that the /s/ sound is susceptible to alteration by the use of a prosthesis [13]. In consonant evaluation, the pronunciation of /s/is generally reported to exhibit a higher sharpness in comparison with other consonants [5]. Therefore, a decrease in the sharpness of /s/, as observed in the current case, implies that the distinct phonetic features have become more challenging to discern and may more readily be confused with other consonantal sounds [4,5]. In this case, subsequent to the adjustment of the prosthesis, sharpness of articulation was observed to converge towards levels observed without a prosthesis, indicating that an articulation closely resembling the natural state had been restored. Clinical speech evaluations typically hinge on the subjective perceptions of the patient and auditory evaluations conducted by clinicians. Nonetheless, applying consonant evaluation to measure speech functions before and after prosthesis adjustment provides a method for the objective assessment of therapeutic efficacy.

This case study implies that adjustments to the prosthesis based on an objective evaluation using an acoustic analysis represent an effective approach for addressing patient-reported speech articulation complaints.

## Conclusions

A patient who had a mandibular defect because of tumor resection received a mandibular prosthesis and experienced speech impairment when it was worn. After the careful adjustment of the prosthesis, supported by an objective, sound analysis of his speech, he was satisfied with the prosthesis. This case suggests that not only maxillary prostheses but also mandibular prostheses may affect speech intelligibility and that consonant evaluation offers an effective, objective method for guiding precise prosthetic adjustments to improve patient outcomes. While the findings are based on a single case, they highlight the need for further research to validate and generalize these observations.
